# Case Report: A simple case of drug-induced secondary antibody deficiency or a rare primary immune deficiency?

**DOI:** 10.3389/fimmu.2025.1629876

**Published:** 2025-11-10

**Authors:** Sarah L. Johnston, Julie C. Evans, Ian R. Berry, Oliver T. Watkinson

**Affiliations:** 1 Department of Immunology and Immunogenetics, North Bristol National Health Service (NHS) Trust, Bristol, United Kingdom; 2 South West Genomic Laboratory Hub, Bristol Genetics Laboratory, North Bristol National Health Service (NHS) Trust, Bristol, United Kingdom; 3 Department of Cardiology, Royal United Hospital, Bath, United Kingdom

**Keywords:** antibody deficiency, primary, secondary, valproate-induced, inborn error of immunity, cardiomyopathy, Roifman syndrome

## Abstract

Antibody deficiency may be primary, because of an underlying inborn error of immunity, or secondary, due to another disease process or medication, leading to decreased antibody production or increased antibody loss. Secondary antibody deficiency is much more common than primary. It can, however, be difficult to distinguish primary from secondary, and both should be considered when assessing patients with hypogammaglobulinaemia. The case presented highlights the importance of confirming the underlying pathophysiology in patients presenting with antibody deficiency, the contribution of genetic assessment to the care of patients with complex phenotypes, and the impact this can have on long-term patient management.

## Introduction

A 41-year-old man, on anticonvulsant therapy, sodium valproate, was referred to the Immunology clinic from the respiratory department of another hospital in 2019, for investigation of immune deficiency, based on a history of recurrent lower respiratory tract infection and borderline hypogammaglobulinaemia.

Examination findings confirmed short stature (height 157.5 cm, below the third centile), diffuse expiratory wheezing on chest examination, but no signs of focal consolidation, a scarred right tympanic membrane, and a grommet in the right external ear canal.

Baseline investigations confirmed a normal total white cell count and differential. Lymphocyte immunophenotyping identified slightly reduced B cells of 87 × 10^6^/L (110–640) accounting for 5% of the total lymphocyte count, but other subsets were normal. Immunoglobulins were borderline with an IgG level of 5.39 g/L (6–16), an IgA of 0.52 g/L (0.8–2.8), and an IgM of 0.86 g/L (0.35–2.42). There was no evidence of a serum paraprotein, serum free light chains were normal, serum albumin level was 38 g/L (35–50), and total protein was 65 g/L (60–80).

The initial working hypothesis was of drug-induced secondary antibody deficiency (SAD), but as the case unfolded, further non-immunological complications led to the identification of a rare primary immune deficiency.

## Case description

The patient was born at term. He was diagnosed with asthma in childhood, requiring frequent hospital admission for management of acute exacerbations until the age of 8 years. He had been under paediatric care in view of poor linear growth until the age of 10 years. He re-presented with recurrent dyspnoea in his teens. The patient was unsure about antibiotic therapy but reported a good response to nebulised bronchodilator. Intensive care support has not been required. When well, he does not have a productive cough but reported having to repeatedly clear his throat. There is no history of haemoptysis. He had recurrent otitis media in childhood, requiring grommet insertion, and reported right-sided hearing compromise as a result. He also reported long-standing sinus congestion but had not needed sinus drainage surgery. There is no history of abscess formation, osteomyelitis, or cutaneous infection. He was unsure about pneumococcal vaccination or when he had last received tetanus vaccination, but had received annual influenza vaccination and standard childhood vaccination according to the United Kingdom vaccination schedule. There is no known family history of primary immune deficiency. In view of the reported lower respiratory tract infection, he was on a trial of doxycycline prophylaxis 100 mg daily at the time of referral. The patient is an ex-smoker of 2 pack years, having stopped 10 years prior to immunological assessment.

Review of data from the other hospital confirmed normal/high immunoglobulin levels in 2008, when undergoing assessment for post-traumatic macroscopic haematuria; IgG level was 7.8 g/L (6–16), IgA 0.91 g/L (0.8–2.8), and IgM 2.0 g/L (0.5–1.9), with a creatinine level of 134 µmol/L. He required admission with dyspnoea in 2011 and had a C-reactive protein (CRP) level of 262 mg/L (<5) at that time, presumed related to lower respiratory tract infection. Plain chest imaging suggested ground glass change possibly secondary to poor inspiratory effort. There were ongoing left basal changes on follow-up chest x-ray, so he proceeded to high-resolution computed tomography (HRCT), which identified widespread bronchial wall thickening, mild bronchiectasis, and distal centrilobular nodules, thought to be inflammatory. Serum angiotensin-converting enzyme and alpha-1 antitrypsin levels were normal; IgE level was 1.7 kU/L (<81). An autoimmune profile, anti-neutrophil cytoplasmic antibody assessment, and *Aspergillus* precipitins were negative. No pathogens were identified on bronchoscopy with bronchoalveolar lavage (BAL) in February 2012, and BAL cellularity data were not available. Transbronchial biopsy confirmed minor focal interstitial chronic inflammation, with no evidence of fibrosis, airspace consolidation, granuloma formation, or neoplasia. Mycobacterial cultures were negative.

Transient low-level microalbuminuria was noted in September 2014, albumin:creatinine ratio was 9.1 mg/mmol (<2.6), and bilateral small kidneys were confirmed on renal ultrasound. Microalbuminuria has been intermittently detected, to a maximum of 12.6 mg/mmol in January 2015; the most recent level was < 3.0 mg/mmol in June 2024. Repeat thoracic HRCT in November 2015 confirmed mild centrilobular bronchiectasis in the lower lobes and lingula.

The patient was seen in the emergency department in February 2017 with respiratory exacerbation and loose stool. Pneumococcal and legionella urinary antigens were negative, as were blood cultures, CRP 97 mg/L, serum folate 1.5 ng/mL (3.0–18.0), and platelets 116 × 10^9^/L. Faecal calprotectin was raised at 204 µg/g. He was treated with antibiotics, but non-infective exacerbation of asthma was on the differential diagnosis. There was minimal interstitial opacification at the lung bases on plain chest x-ray, thought to be inflammatory. Splenomegaly of 15 cm was reported on ultrasound, which has subsequently resolved. Macroscopically upper and lower gastrointestinal endoscopy was unremarkable, and duodenal and colonic biopsies were normal. A short segment of terminal ileal inflammation was identified on subsequent small bowel magnetic resonance imaging (MRI). No intervention was considered necessary. Low-level, intermittent, thrombocytopenia has continued.

Having presented with left hemisensory loss affecting the arm, leg, and left side of the face in May 2017, MRI confirmed a small amount of fluid in the mastoids, but no evidence of cerebrovascular disease. Immunoglobulins in October 2017 confirmed an IgG of 5.8 g/L, an IgA of 0.76 g/L, and an IgM of 1.08 g/L, with no evidence of a paraprotein. Aeroallergen-specific IgEs were negative, and sputum culture was negative. A sweat test was normal in July 2018.

The patient’s other past medical history included gout, chronic kidney disease, mild learning difficulties, epilepsy, anxiety and depression, mild eczema, and a small hiatus hernia. He was on sodium valproate anticonvulsant therapy, which was started in 2007. The family history included ischaemic heart disease (father), cerebrovascular disease, and diabetes. He is an only child and does not have children himself.

At the initial immunology assessment in February 2019, cytomegalovirus and Epstein–Barr virus viral capsid antigen IgGs were positive, Influenzae A complement fixation test titre was 1:64 post-vaccination, of limited value in isolation, but suggestive of a response to either natural infection or vaccination. He had evidence of immunity to measles, mumps, and rubella. *Haemophilus influenzae* antibodies were low at 0.023 µg/mL, with the putative protective level being ≥0.15, and tetanus antibody level was 0.014 IU/mL, with the putative protective level being ≥0.1. On pneumococcal serology, he had protective antibody levels against 1/12 serotypes tested; serotype 19F level was 1.54 µg/mL, with the putative protective antibody level being ≥0.35 µg/mL, in the absence of prior vaccination (confirmed). Human immunodeficiency virus testing was negative. As above, lymphocyte immunophenotyping confirmed borderline low B cells. There was no evidence of thymoma or lymphadenopathy on the 2015 HRCT, interval imaging was stable, and repeat bronchoscopy was negative.

Follow-up was delayed until July 2020 because of the COVID pandemic. In the meantime, the patient had been admitted with dyspnoea in March 2020, with chest imaging confirming bilateral patchy opacification. COVID testing was negative. He responded to steroid and antimicrobial therapy.

Pneumococcal and tetanus vaccination was undertaken with a plan for post-vaccine serology to further assess humoral immune function. A trial of cotrimoxazole prophylaxis was recommended pending the results, based on local practice, with a request to the neurology team to reconsider the sodium valproate, the concern being that this was a potential cause of SAD. Genetic assessment for primary immune deficiency was not considered at this stage.

**Figure 1:**
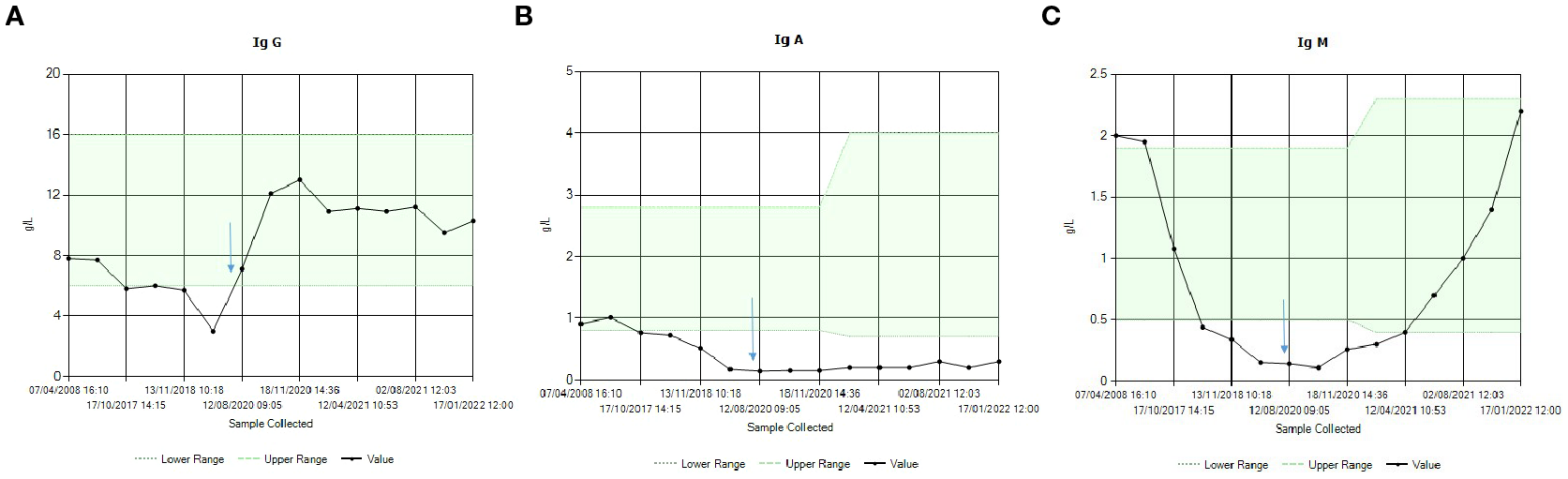
Immunoglobulin levels over time. The blue arrow indicates time of withdrawal of the sodium valproate and initiation of immunoglobulin (IgG) replacement therapy. **(A)** IgG, showing the improvement on immunoglobulin replacement. Over time, the replacement dose has been reduced whilst maintaining a trough level > 8 g/L. **(B)** IgA, showing a normal level in 2008, with minimal improvement following valproate withdrawal. **(C)** IgM recovery following valproate withdrawal.

Progressive panhypogammaglobulinaemia was confirmed from the clinic, with an IgG level of 2.91 g/L, an IgA of 0.2 g/L, and an IgM of <0.1 g/L. Post-pneumococcal vaccination serology confirmed lack of vaccine response, which, given the IgG, was not unexpected. As National Health Service England commissioning criteria for immunoglobulin replacement therapy for SAD were fulfilled (1), immunoglobulin replacement was started in July 2020. This replaces IgG only. Sodium valproate was substituted with lamotrigine, which was complicated by rash, and subsequently switched to lacosamide. There was minimal improvement in the IgA and resolution of the IgM deficiency following valproate withdrawal, with a plan to reduce the replacement immunoglobulin over time if the IgG improved over and above that expected on treatment, depending on the infection burden (**Figure 1**). The patient was keen to continue treatment as his infection and respiratory exacerbation frequency had declined. The primary immune deficiency genetic panel was not requested while SAD was being worked through. Plain chest imaging in January 2022 was clear.

**Figure 2:**
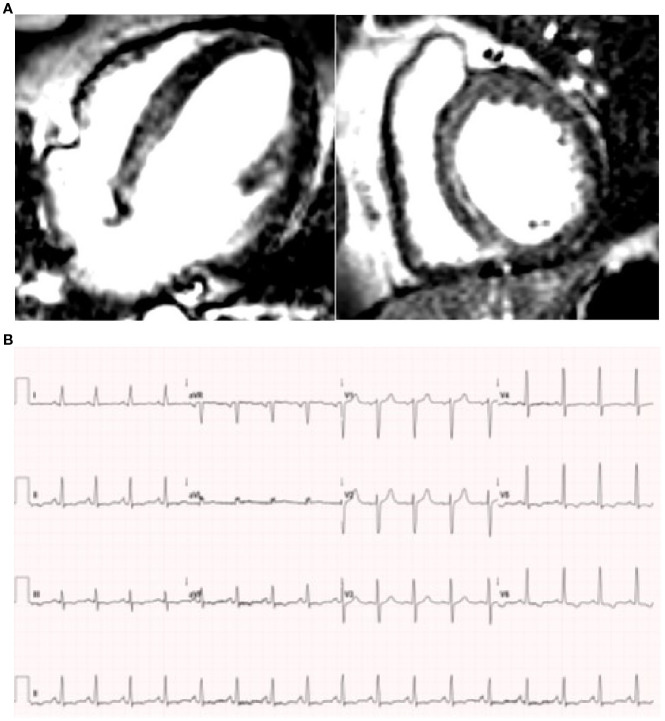
**(A)** Four chamber and short axis slices with late gadolinium from cardiac MRI, showing basal to mid septal mid wall fibrosis. **(B)** ECG showing infero-lateral T wave inversion.

He was re-admitted in April 2023 and treated for atypical pneumonia in view of raised inflammatory markers (CRP 95 mg/L) and CT pulmonary angiography showing extensive ground glass change, with the differential diagnoses of infection and pulmonary oedema. Respiratory viral polymerase chain reaction (PCR), pneumococcal and legionella urinary antigens, serum beta D glucan, and procalcitonin were negative. Symptoms were refractory to antimicrobial therapy. Troponin I was 23 ng/L (<20) and N-terminal proB-type natriuretic peptide was 4,709 ng/L (<400 ng/L), prompting urgent cardiology assessment. Non-ischaemic cardiomyopathy was then confirmed on MRI, the left ventricle (LV) being relatively dilated compared to the right, with severe LV impairment and mid-myocardial septal fibrosis. A possible small mid-LV inferolateral infarct was suggested (**Figure 2**). Coronary angiography was normal. Introduction of heart failure therapy was complicated by an acute kidney injury, with a maximum creatinine of 242 µmol/L. Renal artery stenosis was subsequently excluded on imaging.

Genetic testing by whole genome sequencing given the clinical phenotype of dilated cardiomyopathy, learning difficulties, epilepsy, short stature, and immunoglobulin deficiency confirmed a compound heterozygous status for a pathogenic *RNU4ATAC* variant and a likely pathogenic *RNU4ATAC* variant, consistent with a genetic diagnosis of *RNU4ATAC*-associated Roifman syndrome.

Following appropriate management of the cardiomyopathy, the patient has been well. Moraxella has been confirmed on sputum culture on two occasions over the last 12 months. Given the diagnosis of Roifman syndrome, the plan is to continue the immunoglobulin replacement.

## Discussion

SAD, characterised by reduced antibody levels because of another disease process or medication, leading to decreased antibody production or increased antibody loss, is much more common than primary antibody deficiency due to an underlying inborn error of immunity. SAD is being increasingly recognised as a result of medical therapies, particularly those targeting B lymphocytes, for example, in the management of haematological malignancy and systemic autoimmune disease. It can be difficult to distinguish primary antibody deficiency from SAD, and both should be considered when assessing patients with hypogammaglobulinaemia (2).

The initial concern in this case was of sodium valproate-induced antibody deficiency, noting that the patient’s immunoglobulins were normal at around the time sodium valproate was started. Valproate-induced panhypogammaglobulinaemia has previously been described (3). Other sodium channel-blocking anticonvulsants, including phenytoin and carbamazepine, have also been implicated in symptomatic reversible antibody deficiency (4–6). The initial improvement in the IgM following drug withdrawal supported this. The low-level, intermittent microalbuminuria was considered unlikely to explain the antibody deficiency, especially given the low IgA and IgM. Hyper immunoglobulin IgM syndrome was not considered as the normal immunoglobulins in 2008 did not suggest a class-switch recombination defect.

Following the diagnosis of the cardiomyopathy, the clinical phenotype was reconsidered. The constellation of dilated cardiomyopathy, learning difficulties, epilepsy, short stature, bronchiectasis, and immune deficiency prompted whole genome sequencing, with application of the relevant Genomics England panels (paediatric or syndromic cardiomyopathy; primary immunodeficiency or monogenic inflammatory bowel disease; and paediatric disorders). Compound heterozygous status for a pathogenic *RNU4ATAC* variant *RNU4ATAC* NR_023343.1:n.13C>T and a likely pathogenic *RNU4ATAC* variant *RNU4ATAC* NR_023343.1:n.13C>G, consistent with a genetic diagnosis of *RNU4ATAC*-associated Roifman syndrome, was confirmed on the paediatric disorders (45.2) panel. Both variants have previously been reported.

Roifman syndrome is a rare congenital syndrome, originally characterised by recurrent infection due to antibody deficiency, spondyloepiphyseal dysplasia, growth retardation, and retinal dystrophy. It was first described in a four-patient case series by Roifman in 1999 (7). All four patients were reported to be of short stature, with slightly short hands and feet, and clinodactyly of the fifth finger was seen in all four. Whilst it was originally thought to be an X-linked condition, as the initial reports were of male patients, and non-random X-inactivation was reported in a female case with a younger brother with clinical features of Roifman syndrome (8), it was subsequently confirmed by whole genome sequencing to be an autosomal recessive disorder caused by compound heterozygous mutations in the non-coding *RNU4ATAC* gene (9). The rare variants described disrupt highly conserved positions of the *RNU4ATAC* small nuclear RNA gene, a minor spliceosome component that is essential for minor intron splicing. Whole exome sequencing will not identify such mutations as they are located in noncoding regions. Further mutations have been described, including homozygous mutations in the stem II region, which is sufficient to cause the full spectrum of features associated with Roifman syndrome (10). Two siblings with early-onset primary antibody deficiency resembling common variable immunodeficiency were described in 2017 (11), found by targeted genetic sequencing.

Mutations in *RNU4ATAC* have also been reported in microcephalic osteodysplastic primordial dwarfism type 1 (MOPD1) (9, 10) and Lowry-Wood syndrome (12), leading to the designation of *RNU4ATAC* Spectrum Disorder or RNU4atac-opathy (13), encompassing the three conditions. Common findings include growth restriction, microcephaly, skeletal dysplasia, and cognitive impairment. More variable findings include brain anomalies, seizures, immune deficiency, cardiac abnormalities, ophthalmic, cutaneous, renal, gastrointestinal, hearing, and endocrine involvement. MOPD1 is usually fatal in the first year of life; Roifman syndrome and Lowry-Wood syndrome are typically less severe.

To date, 15 cases of Roifman syndrome have been reported in the medical literature, 13 until 2018 (14), with the 2 most recent cases being brothers with characteristic skeletal abnormalities (15). A further case has been described with overlapping features of the three reported RNU4atac-opathies (16).

Cardiomyopathy has been described in two patients with Roifman syndrome. The first reported case was of noncompaction of the ventricular myocardium, with prominent trabeculation and deep intratrabecular recesses within the ventricular wall (17). The second reported case was a 20-year-old man, with microcephaly, retinitis pigmentosa, recurrent ear infection, and endocrinopathy. Having presented with exertional dyspnoea and signs of heart failure, investigation confirmed non-specific, non-ischaemic cardiomyopathy (18).

In addition to the reported skeletal abnormalities, cognitive and behavioural abnormalities have been described, including learning difficulties and neuropsychological impairment (19). In one case, partial agenesis of the corpus callosum and hippocampal atrophy were identified on MRI with reportedly stable intellectual disability (20).

**Figure 3:**
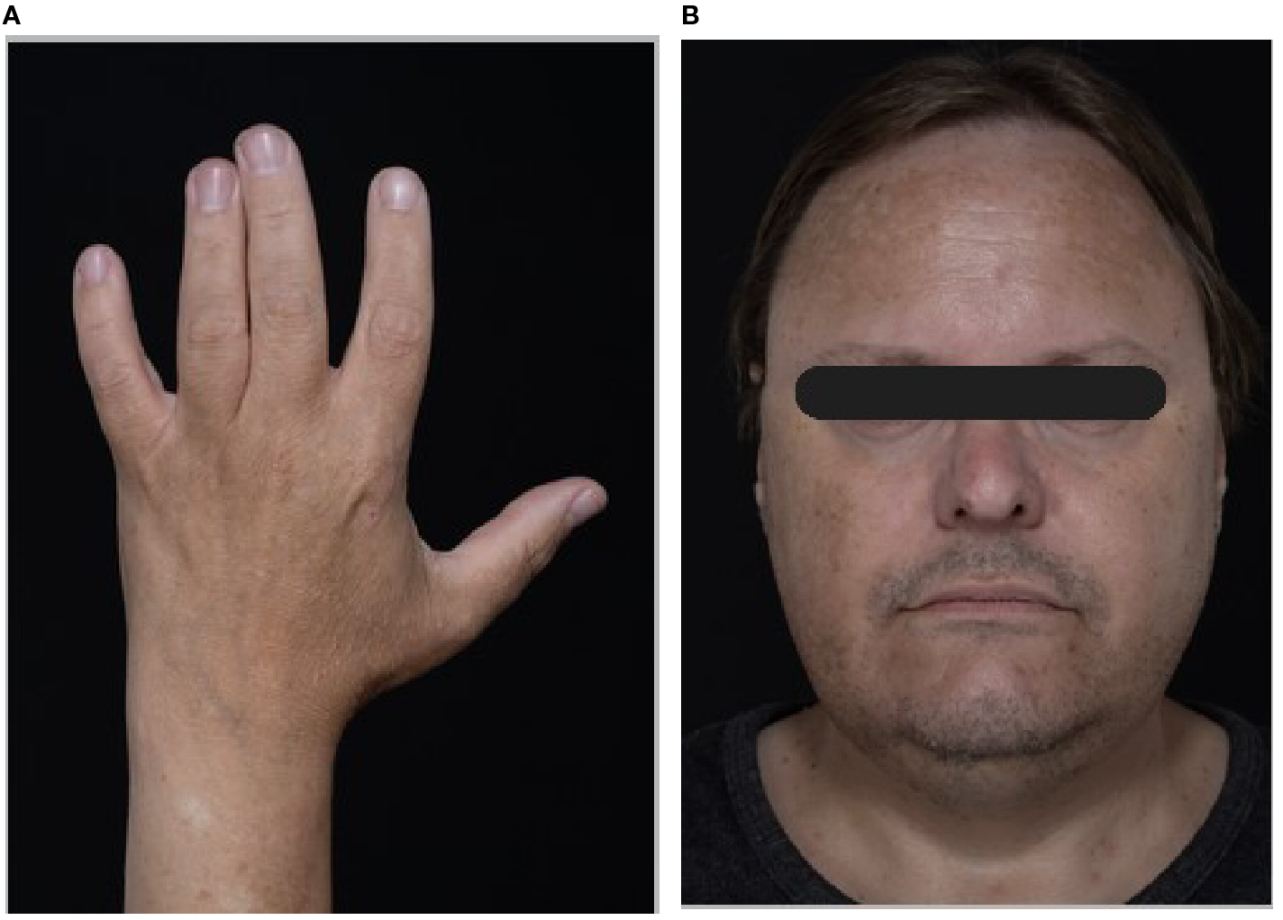
Patient images. **(A)** Patient’s left hand showing brachydactyly and subtle fifth finger clinodactyly. **(B)** Facial image confirming thin upper lip as reported in Roifman syndrome (7).

Whilst our patient had subtle features of Roifman syndrome, including short stature, mild learning difficulties, epilepsy, and, in retrospect, mild brachydactyly and subtle clinodactyly (**Figure 3**), he was originally thought to have drug-induced antibody deficiency. It was not until he presented with non-ischaemic cardiomyopathy at the age of 45 that a unifying diagnosis was reached on genetic analysis. All available radiology has been reviewed, including cerebral MRI. Apart from the brachydactyly, there are no confirmed skeletal features of Roifman syndrome and no evidence of corpus callosum or hippocampal pathology. The patient does not have microcephaly. Hearing loss is consistent with otitis media, and he has refractive eye disease but no confirmed retinal dystrophy and there has been no endocrinopathy to date.

One of the two genetic variants described, the *RNU4ATAC* NR_023343.1:n.13C>T variant, is considered to have a damaging effect, with functional studies showing that this variant reduces splicing efficiency to 60%–80% of wild type (21). The second variant, *RNU4ATAC* NR_023343.1:n.13C>G (occurring at the same nucleotide position), falls within the stem II domain known to be important for splicing and a key hot spot of Roifman syndrome variants (9). The relatively mild phenotype of our case may be related to residual activity of at least the 13C>T variant. It is unknown at this stage what the effect of the 13C>G mutation has on splicing function. Whilst the patient is the oldest reported case at diagnosis, it may be that more cases will be identified as genetic testing is increasingly applied.

**Figure 4:**
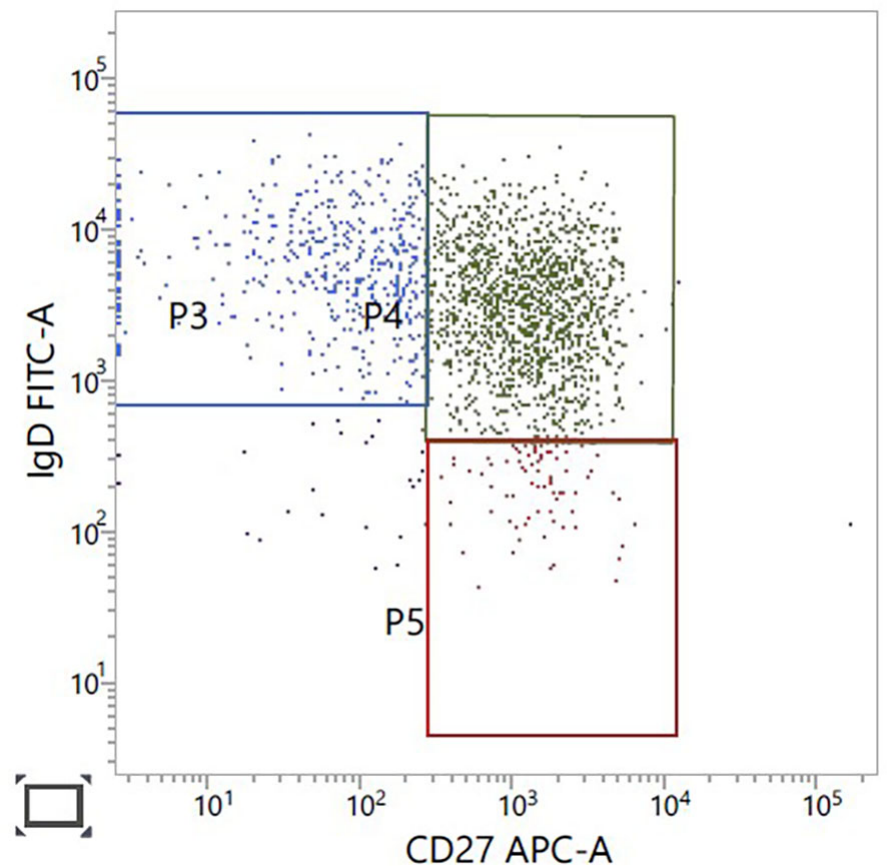
B-cell immunophenotyping gated on CD19, CD27-positive B cells. CD27-positive, IgD-positive non-class switched memory B cells in green, upper right panel.

The patient’s cardiac condition has stabilised on treatment. He continues taking lacosamide for the epilepsy. There have been no recent generalised seizures. With respect to immune deficiency, further lymphocyte immunophenotyping in 2024 confirmed normal B-cell numbers, 260 × 10^6^/L (110–640), but the majority of the patient’s B cells are non-class-switched memory cells (**Figure 4**). No distinct class-switched memory B-cell population was identified. B-cell lymphopenia, reduction in class-switched memory B cells, and differentiation block at the transitional B-cell stage have all been reported (15, 22).

The contribution, if any, of sodium valproate to the antibody deficiency remains uncertain. With the genetic findings, limited IgA recovery following sodium valproate withdrawal, immunophenotyping, and the recently confirmed breakthrough bacterial infections, immunoglobulin replacement therapy is set to continue. The patient has been referred to, but has, to date, declined, clinical genetic assessment, but he remains well under immunology, cardiology, and neurology follow-up.

## Conclusion

This case highlights the importance of differentiating primary from secondary immune deficiency, which is not always straightforward (2), and the contribution of genetic assessment to the care of patients with complex phenotypes.

## Data Availability

The authors acknowledge that the data presented in this study has been deposited and is publicly available in the following hyperlinks: "https://www.ncbi.nlm.nih.gov/clinvar/variation/218083/"VCV000218083.46 - ClinVar - NCBI "https://www.ncbi.nlm.nih.gov/clinvar/variation/2151940/"VCV002151940.2 - ClinVar - NCBI.
